# Scale-free statistics of neuronal assemblies predict learning performance

**DOI:** 10.1186/1471-2202-12-S1-O4

**Published:** 2011-07-18

**Authors:** Petra E Vertes, Danielle S Bassett, Thomas Duke

**Affiliations:** 1 Cavendish Laboratory, University of Cambridge, Cambridge, CB3 0HE, UK; 2 Brain Mapping Unit, Department of Psychiatry, University of Cambridge, Cambridge, CB2 3EB, UK; 3 Department of Physics, University of California, Santa Barbara, CA 93106, USA; 4 London Centre for Nanotechnology, University College London, London, WC1H 0AH, UK

## 

Recent years have seen a profusion of research and controversy surrounding the hypothesis that the brain may be operating at criticality. While experimental evidence supporting the presence of scale-free avalanches in neuronal activity characteristic of a critical state has been steadily accumulating, the interpretation of these data as evidence for critical brain dynamics has simultaneously been questioned (see [1,2] and references therein). From a neuroscientific point of view, the principal appeal of the criticality hypothesis is that critical dynamics have been repeatedly linked to improved information processing, including increased memory capacity, dynamic range, and computational power (see [2] and references therein). Thus far, however, the role that neuronal avalanches - whether hallmarks of criticality or not - might play in complex mental processes such as learning and memory has remained largely unexplored.

In this paper, we show that scale-free cascades of neuronal activity also characterize artificial neural networks performing Hebbian learning tasks [3,4]. Our experimental framework enables us to simultaneously measure the network’s performance on the task while observing the corresponding size-distribution of neuronal assemblies. These phenomena are assessed as a function of both network topology and the degree of excitation versus inhibition.

Smoothly tuning the degree of order in the network topology, we find that task-performance is optimized in the dynamical state associated with scale-free cascades of exponent 2 < α < 3 which occur in so-called ‘small-world’ networks characterized by high local clustering and short path-length. A transition to ordered, lattice-like, networks is accompanied by both the sudden onset of severely impaired performance and the loss of scale-invariance in neuronal responses. Furthermore, we find that this correlation between scale-free neuronal cascades and performance also holds true when variations in performance are achieved by altering the network’s excitatory-inhibitory balance. Performance is optimized in balanced networks, whilst excitation-dominated states are characterized by a simultaneous drop in performance and the loss of scale-invariance. This is in keeping with experimental studies in cortical slices where the application of picrotoxin, a GABA-receptor antagonist that reduces inhibitory activity, has been shown to result in the loss of power laws in local field potential recordings [5].

Finally, we note that the performance of the system is more robust to deviations from excitatory-inhibitory balance in small-world networks than in random ones. These results may provide insight into psychiatric diseases such as schizophrenia where decreased inhibitory activity [6] and a disruption of topological organization [7] have both been observed alongside the well-known cognitive impairments associated with the disease.
